# The Hypersensitive Response to Plant Viruses

**DOI:** 10.3390/v15102000

**Published:** 2023-09-26

**Authors:** Maïlys Piau, Corinne Schmitt-Keichinger

**Affiliations:** UMR_A1131 Université de Strasbourg-INRAE, 68000 Colmar, France; mailys.piau@inrae.fr

**Keywords:** LRR proteins, LRR-RLP, LRR-RLK, NB-LRR, defense response, virus, extreme resistance, systemic necrosis, HR and HR-like, *R* gene, programmed cell death

## Abstract

Plant proteins with domains rich in leucine repeats play important roles in detecting pathogens and triggering defense reactions, both at the cellular surface for pattern-triggered immunity and in the cell to ensure effector-triggered immunity. As intracellular parasites, viruses are mostly detected intracellularly by proteins with a nucleotide binding site and leucine-rich repeats but receptor-like kinases with leucine-rich repeats, known to localize at the cell surface, have also been involved in response to viruses. In the present review we report on the progress that has been achieved in the last decade on the role of these leucine-rich proteins in antiviral immunity, with a special focus on our current understanding of the hypersensitive response.

## 1. Introduction

Viruses represent a major threat to crop plants and global food security [1], which will most likely be further exacerbated by climate change and human population pressures [2,3]. This huge challenge will require integrated, knowledge-based management of plant virus epidemics. Only combinations of measures are believed to provide efficient disease control because they act at different levels and pathways [3,4]. Among these measures, natural and engineered resistances are promising, provided that they are founded on robust knowledge and long-term experiments under field conditions. To fight pathogens, plants rely on an innate immune system based on a few defense mechanisms that will either become decisive or be circumvented in an arms race against the pathogens. 

Because plant viruses entirely depend on cellular processes, some plants with allelic variants of host factors that do not interact with the virus present a recessive resistance [5,6,7]. This type of resistance opens great perspectives as virtually any newly identified host factor required for virus multiplication becomes a potential source of resistance if a loss-of-interaction mutant is identified or engineered. Another important kind of resistance originates from RNA silencing [8,9] which has been discovered more recently but whose mechanisms have been rapidly unveiled. It is triggered by double-stranded RNA (dsRNA) and involves Dicer-like (DCL) endonucleases to generate small viral RNAs (vsRNAs) capable of guiding an RNA-induced silencing complex (RISC) for the degradation, or the translation inhibition, of long RNAs or for the transcription inhibition of DNA viruses [8,9,10,11]. This mechanism underlies parasite-derived resistance, a general strategy deriving resistance genes from the pathogen’s genome [12,13,14]. A better comprehension of this mechanism has allowed the deployment of hairpin- and artificial microRNA-producing genes to confer resistance to potentially any plant virus [14,15,16,17,18,19] with very limited production of pathogen-derived RNA and hence limited risks of hetero-encapsidation or recombination [13]. 

**Figure 1:**
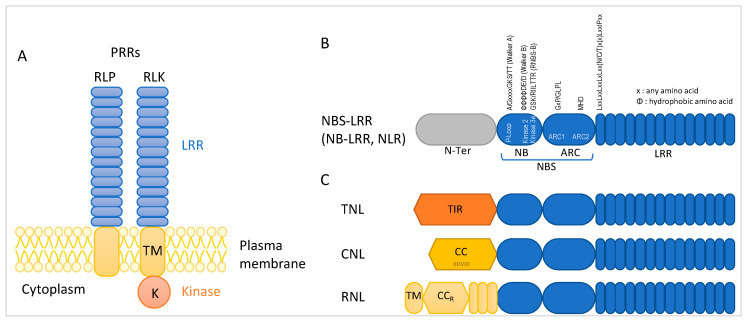
Schematic representation of LRR proteins involved in plant immunity. (**A**). Receptor-like proteins (RLPs) and receptor-like kinases (RLKs) are located at the cell surface. (**B**,**C**). NBS-LRR proteins are cytoplasmic. (**A**). Domains are depicted and their designation is indicated under the diagram, subdomains are identified in the forms, and consensus sequences of the motifs are specified above the chart. The single letter code is used to represent amino acids. (**C**). Three classes of NBS-LRR proteins are distinguished depending on their N-terminus: TNL possess a domain with homology to *Drosophila* Toll and human Interleukin 1 receptors (TIR), CNL have a classical coiled coil domain, and RNL resemble RPW8 (Resistance to powdery mildew 8) with a potential transmembrane domain (TM), a specific coiled coil domain of RPW8 type (CC_R_) and RPW8 repeats.

**Figure 2:**
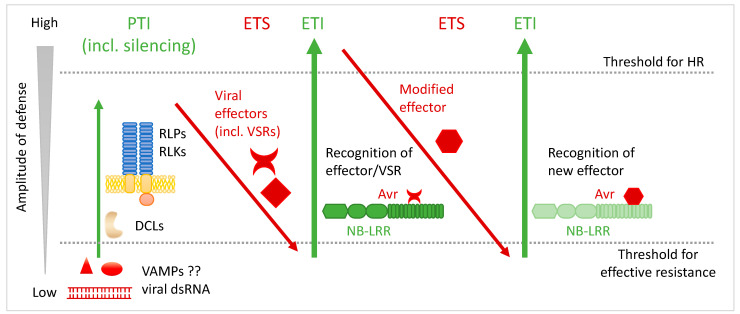
The zig-zag model illustrates the arms race between plants and viruses. Silencing is often seen as pattern-triggered immunity (PTI) with DCLs recognizing viral dsRNAs. PTI is also involved in antiviral defense in a more classical way, through pattern recognition receptors (PRRs) independently of silencing. This first layer of resistance is considered weak and can be overcome by viral effectors, including viral suppressors of RNA silencing (VSRs). This results in effector-triggered susceptibility (ETS). A second layer of resistance is based on the direct or indirect specific recognition of viral effectors (Avr factors) by *R*-encoded NB-LRR proteins, leading to an effector-triggered immunity (ETI) which is considered an amplified version of PTI if it is rapid and strong enough to induce hypersensitive cell death (HR). Virus isolates can emerge with modified effectors that escape recognition. In turns, plants can evolve new NB-LRRs able to recognize the new effector, regaining ETI. VAMP: virus-associated molecular pattern, like viral dsRNA or other viral factors. Adapted from [26] and from [23].

Another level of plant resistance is conferred by dominant genes mainly encoding proteins with domains rich in leucine repeats (LRR). In a first layer, pathogen-associated molecular patterns (PAMPs) are recognized by membranous proteins at the cell surface: pattern recognition receptors (PRRs, Figure 1A). They possess an extracellular LRR domain and are either with no structured internal domain and are called receptor-like proteins (RLPs) or with a kinase domain and are called receptor-like kinases (RLKs). Upon ligand binding, many PRRs associate with RLKs, leading to a rapid resistance to a broad range of pathogens [20]. This pattern-triggered immunity (PTI) was long considered restricted to bacteria, fungi, and oomycetes [21,22] because of the direct entrance of phytoviruses within the cell, confining PTI against viral pathogens to animal viruses. The concept of PTI was later broadened to include plant viruses and RNA silencing: dsRNA was seen as a pathogen- or virus-associated molecular pattern which is detected by DCLs and followed by a defense response [23]. RNA silencing has more recently been considered a kind of adaptive immunity of plants [24,25]. PAMPs are generally conserved throughout classes of pathogens (like flagellin in bacteria and chitin in fungi) and PTI is described as a weak basal defense that can be overcome by pathogen-encoded effectors, including secreted bacterial or fungal effectors and viral suppressors of RNA silencing. This gave rise to the so-called zig-zag model [26] and modified zig-zag model for viruses [23] where the circumvention of the first layer of defense is followed by a second layer, based on effector recognition, essentially by intracellular proteins (Figure 1B and Figure 2). These proteins also containing LRR domains trigger a race-specific reaction reported to be quicker, more robust, and exceeding the threshold of programmed cell death (PCD). This reaction is referred to as hypersensitive response (HR) [23,26,27,28,29]. This effector-triggered immunity (ETI) results in the restriction of the pathogen at its entry site. However, it can be overcome by evolved pathogen isolates escaping recognition, and plant genotypes in turn may evolve new receptors to recognize modified or new effectors. This dichotomic distinction between PTI represented in the plant virus field by RNA silencing and ETI leading to HR is being increasingly undermined by recent advances in specific pathosystems that revealed a link between plant viruses and PTI independently of RNA silencing [20,30,31,32]. Moreover, HR as an antiviral defense against viruses has been revisited during the last decade and is now rather seen as being at some point along the continuum of plant–virus interaction ranging from extreme resistance (ER) to total susceptibility [33]. In this review we report on recent advances in plant immunity against viral infections with a special focus on the evolution of our knowledge on LRR proteins, the concept of HR, and its regulation by ubiquitin and autophagy.

## 2. Resistance Genes to Viral Infection

### 2.1. Involvement of LRR-RLK Encoding Genes in Response to Viral Infection

Although viruses are intracellular parasites, several lines of evidence have emerged in the last decade showing that LRR-RLK proteins, mainly located at the cellular surface, play a role in the response to virus attacks [34]. In 2013, a seminal work showed that the response of *Arabidopsis thaliana* to turnip crinkle virus (TCV) infection depends on BAK1 (also named SERK3), an RLK known to interact with both the flagellin receptor FLS2 and the peptide receptors PEPRs sensing the production of small peptides after wounding [30]. Neither the PRR nor the elicitor of the response were identified but this was the first robust evidence that plant viruses can induce PTI. A similar genetic demonstration that known PTI actors contribute to immunity to plum pox virus (PPV) was completed by the identification of PPV CP as a suppressor of early PTI [32]. In 2016, Niehl and colleagues demonstrated that Arabidopsis plants can perceive dsRNA or its analog poly(I:C) to induce PTI responses independently of *DCLs*, suggesting that dsRNAs represent authentic PAMPs that are recognized through complexes containing the BAK1-associated SERK1 RLK [31]. At the same time, protein P6 of the DNA virus cauliflower mosaic virus (CaMV) was shown to suppress hallmarks of PTI-like ethylene production and extracellular reactive oxygen species (ROS) burst induced by the flg22 elicitor [35]. A dsRNA binding motif of P6 was necessary for PTI suppression but dispensable for the silencing suppression activity, proving that these two activities are uncoupled. In the case of begomoviruses in the family *Geminiviridae*, it was shown that viral DNAs and RNAs, if applied by rubbing, induce an antiviral response through the LRR-RLKs NIK1 and NIK2 belonging to the SERK clade. Autophosphorylation of these RLKs leads to phosphorylation of RPL10 and global inhibition of the translation machinery [20]. Although not involving the classical signaling of BAK1-dependent PTI, this pathosystem clearly involves cell surface localized LRR-RLKs and recognition of viral PAMPs. Whether this recognition occurs extracellularly following the release of PAMPs through wounding or intracellularly via the kinase domain, and whether NIKs represent receptors or co-receptors, remain to be elucidated [20]. Since then, other viral suppressors of PTI have been identified, including proteins Rep and C4 from geminiviruses [36], the MP of cucumber mosaic virus (CMV) [37], the begomovirus-associated satellite ßC1 protein [36], or the CPs of necroviruses [38], although in the two latter cases, viral effectors act in the mitogen-activated protein kinase (MAPK) cascades that are rapidly activated in PRR signaling and undergo a slower but longer-lasting activation in ETI [39,40], making it difficult to identify the inhibited pathway. Altogether these works identified (i) viral nucleic acids (dsRNA, DNA or RNA) as elicitors, (ii) known LRR-RLK localized at the cell surface as receptors or co-receptors of viral PAMPs, and (iii) viral effectors able to suppress PTI. In addition, a number of genetic, transcriptomic, and proteomic analyses have identified LRR-RLKs and other PTI actors in response to diverse viruses or viral protein overexpressed in different plant species [41,42,43,44,45,46], further generalizing the role of LRR-RLKs and classical PTI in plant antiviral immunity.

### 2.2. NBS-LRR Encoding R Genes

The second layer of plant defense to pathogens is operated in a gene-for-gene relation first proposed by Flor [47]. This concept suggests that for each gene that conditions reaction in a host there exists a corresponding gene in the parasite that conditions pathogenicity. This is embodied by the specific interaction between the product of a resistance gene (*R*) encoded by a plant genotype and the avirulence factor (Avr) encoded by a given strain of the parasite [48]. This race-specific interaction leads to defense reactions and resistance and is qualified as incompatible relation, whilst an absence of interaction and resistance leads to a compatible relation where the parasite colonizes its host [49]. Both *R* and *Avr* genes are dominantly inherited. The pathogen alleles escaping recognition and thus leading to a compatible reaction, also known as virulence genes, are recessive. Similarly, alleles of the *R* gene encoding proteins that do not recognize the Avr factor are recessive. 

The first cloned *R* gene was the *N* gene conferring resistance to tobacco mosaic virus (TMV). Cloning used a maize activator transposon to induce mutations and took advantage of the reversible thermosensitivity of the N protein to turn the search for loss-of-function mutants (TMV-susceptible) into a positive selection [50]. The protein deduced from the cloned sequence contained a nucleotide binding domain (NB) encompassing the three classical motifs: (i) the P-loop binding the phosphates of ATP or GTP (consensus sequence A/GXXXXGKS/T), (ii) the kinase 2 motif defined by four consecutive hydrophobic residues followed by a conserved aspartic acid (D) which coordinates Mg^2+^ cations, and (iii) the kinase 3a motif involved in purine or ribose binding, and a LRR domain containing 14 LRRs each composed of approximately 26 amino acids [50]. These two domains have since been observed in the vast majority of cloned *R* gene products (Figure 1B). An additional domain has been described between NB and LRR called the ARC domain, where ARC stands for APAF-1 (human apoptotic peptidase-activating factor 1), R (plant resistance protein), and CED4 (cell death abnormal 4 of *Caenorhabditis elegans*), all playing a role in PCD. ARC is further subdivided into subdomain ARC1 containing a GxP or GLPL motif and ARC2 containing an MHD motif [21,51,52,53]. Both NB and ARC are thought to contribute to nucleotide binding and hydrolysis and are thus together termed the NBS (nucleotide binding site). These proteins are collectively called NBS-LRRs. Alternate names are NB-LRRs and NLRs for NOD (nucleotide-oligomerization domain)-like receptors because NB-ARCs are structurally similar to NOD domains. Depending on their N-terminal domain, two main classes were first described: TIR-NBS-LRR (TNL) proteins possess a Toll/ Interleukin-1 Receptor like (TIR) domain and CC-NBS-LRR (CNL) proteins have a coiled-coil (CC) N-terminal domain, TNL encoding genes being only present in dicots [53]. Later, proteins with a specific CC domain (noted CC_R_) devoid of the classical EDVID motif and similar to the CC domain of RPW-8 (Resistance to powdery mildew 8) of Arabidopsis were distinguished and called RNLs. They contain an N-terminal potential transmembrane domain and C-terminal repeats of unknown activity [54,55,56]. 

Many *R* genes have been cloned from many different plant species, among which 26 confer resistance to viruses (Table 1). Cloning and identifying *R* genes remains a time-consuming and laborious task. It mostly relies on marker-assisted mapping in experimental crosses between susceptible and resistant parents [57]. Resistant parents either originate from natural populations or from crops where the *R* gene was previously introgressed. Although co-segregating markers are diverse (microsatellites, RFPLs, RAPDs, SNPs, etc.) and positioned at high densities, some resistance genes/loci still await cloning and confirmation of their function. This is the case for resistances discovered many years ago, such as the potato (*Solanum tuberosum*) genes *Ny* or *Nc* and *Nb* conferring resistance to PVY and PVX, respectively, or the *I* locus of common beans (*Phaseolus vulgaris*) [58], and also for more recently discovered resistance sources such as *Ry(o)_phu_* in potato or *Cbd* and *Cbt* in *Gossypium hirsutum* [59,60,61,62]. Like for resistance toward non-viral pathogens, cloning efforts have benefitted from high throughput sequencing in both genomic and transcriptomic studies [63]. Resistance gene enrichment sequencing (RenSeq) and single-molecule real-time sequencing (SMRT) allowing long reads after capture of NLR encoding sequences [64,65] were applied to identify the potato *Ry_sto_* resistance gene (Table 1) [66]. Resistance genes can also be identified through the loss of their function (loss of HR phenotype), as was originally done using transposon tagging [50,67]. More recently, a library of hairpins was used in a proof-of-concept experiment [68]. Virus-induced gene silencing followed by the use of a library of guide RNAs in a Cas 9-based KO approach allowed the cloning and confirmation of the soybean *Rsc 4-3* resistance gene to soybean mosaic virus [69,70]. Conversely, a gain-of-function approach was employed for the identification of the potato *Rx-2* gene [71] and the pepper gene *Prv9* [72,73]. For that, a library of *R* gene homologues needs to be cloned in a transient agrobacterium-mediated expression system. The virus or viral Avr factor (if known) and the candidate *R* genes are expressed in a compatible host, which is then screened for the expression of the HR phenotype. This is a rather straightforward method, which has also been widely used for the final demonstration of the functionality of many *R* genes isolated by mapping or homology cloning, as an alternative to transgenesis in susceptible plants [66,70,73,74,75,76,77]. There is no doubt that a combination of all these techniques will increase the pool of cloned resistance genes to plant viruses in the few next years.

## 3. Avr Factors and Avr–R Recognition Models

Identification of viral Avr factors is easier and quicker, provided that an inoculation procedure or a transient expression system is working on the resistant plant. An observation of the cell-death phenotype usually allows for rapid screening of the few viral candidate sequences. Knowing avirulent and virulent isolates can greatly help in designing these candidates through sequence comparison or construction of chimeric viruses (Table 1). Transient expression systems include agrobacterium infiltration or viral vectors. The use of chimeric viruses or even heterologous viruses has the advantage of allowing assessment of not only the cell-death response but also the virus restriction, as well as the symptom reduction in non-inoculated leaves. These approaches have led to the characterization of most cognate Avrs of known resistance genes to plant viruses (Table 1) [70,73,78,80,84,91,94,100,103,106] and also to the identification of a lot more viral Avrs, even with no or poor knowledge on the corresponding *R* gene, as exemplified by the 25 kDa MP of PVX eliciting the *Nb* potato gene [110], P0 of different poleroviruses eliciting the *RPO1* allele of *N. glutinosa* [111] and the *Cbd* and *Cbt* genes in cotton [111,112], P6 of CaMV in *Nicotiana edwardsonii* [113], a nepovirus 2A^HP^ in *N. occidentalis* [114], the tomato spotted wilt virus (TSWV) NSm in *N. alata* [115], or a geminivirus RepA in *N. benthamiana* [116]. Information for the identification of viral Avr candidates also arise from the emergence of resistance breaking isolates [84,106,117,118]. Due to their efficiency and ease of implementation, these techniques have barely evolved over the years.

The recognition of Avr factors by *R*-encoded products has been largely studied and reviewed [119,120,121]. The C-terminal LRR domain of R proteins has been shown to determine the specific recognition of Avrs. This was primarily shown by domain-swapping experiments and mutational analyses [74,76,122]. Although this corresponds to the general case, the TIR domain of the *N* gene has been involved in the specific recognition of the TMV helicase domain [123]. This could participate in a two-step recognition mechanism, as proposed for the Sw5-b where the LRR and the N-ter domains both interact with NSm to allow the detection of low levels of NSm and induce a robust HR [124].

**Figure 3:**
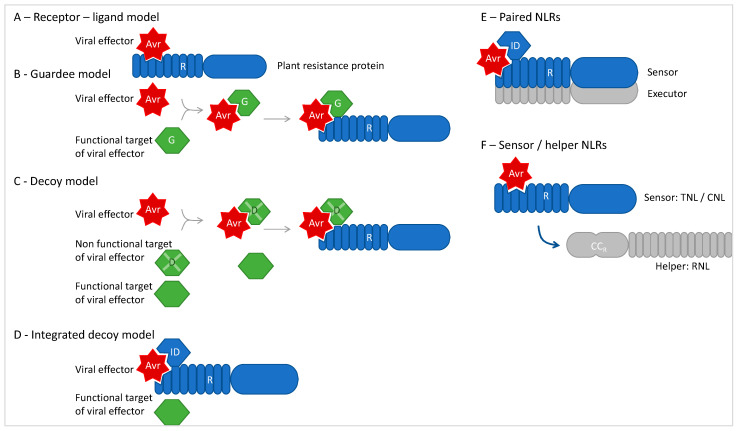
Models of NB-LRR (R)–Avr recognition. (**A**). Direct recognition of the viral Avr by the plant NB-LRR protein. (**B**). NB-LRR surveys the plant target of the viral effector and detects its modification by the Avr binding. This host target becoming a cofactor of the recognition is termed a “guardee” (G). (**C**). Selection may favor plant factors with decreased or no virulence that mimic the actual virulence target. These so-called decoys (D) thus act as molecular sensors of the pathogen. (**D**). Some NB-LRR proteins have integrated domains (IDs) that are essential in effector recognition. (**E**). ID-NLRs can act in paired NLRs: they act as sensors, inhibiting the associated executor NLR in the absence of Avr. (**F**). In the sensor/helper pair, the helper is induced after the sensor has detected the Avr but there is no physical link between the sensor and the helper.

Different models of R-Avr recognition have evolved to explain the detection of the virus’ presence by the R protein (Figure 3). The virus can be detected through a direct interaction between Avr and R. Such a direct interaction has been evidenced between the tomato *Sw5-b*-encoded NLR and the American type of tospovirus cognate NSm Avrs [125]. Direct interactions have also been shown to be possible between the Sw5-a protein and the ToLCNDV-encoded AC4 [108] and between the soybean Rcs 4-3 NLR and the potyvirus CI protein [70]. Indirect interactions, requiring a plant cofactor, can also account for virus detection following either the guardee model or the decoy model or the more recently described integrated decoy model [119,126,127,128]. In the guardee model, the R protein detects the virus’ presence through the high-jacking of the Avr’s cellular target; the R protein surveys or guards the target that is termed guardee. Because host targets perform essential functions, plants have evolved non-functional targets that act as decoys and have increased abilities to evolve. Finally, some decoys seem to have fused with R proteins. Such additional domains contained in R proteins are called integrated decoys (IDs), and the R–Avr recognition follows the integrated decoy model (Figure 3). Among proteins interacting with antiviral NLRs, the RanGAP2 protein of potato interacting with Rx was hypothesized to act as a guardee [129,130]; however, no interaction with the Avr PVX CP could be proved, unlike the chloroplastic NRIP1, which interacts with both the p50 Avr and N TNL [123]. Sun and colleagues have recently listed seven kinds of NLR-interacting proteins and proposed that guardees and decoys rather belong to kinase or pseudokinase families [131]. Antiviral NLRs with supplemental domains that could fit the integrated decoy model are scarce, although this type of NLR is widespread in plant genomes [128]. They include PvVTT1 which contains a second TIR domain [90], Sw5-b with an extended N-terminal domain referred to as the *Solanaceae* domain (SD) shown to contribute to Avr recognition [124], and the melon Prv containing an additional NBS domain in its C-terminus [97]. Many ID-containing NLRs (ID-NLRs) act in a pair with a second NLR [127]; the ID-NLR is the sensor, whereas the second, a canonical NLR, is the executor acting as a signal transducer. The best known examples are the RGA4/RGA5, Pik-1/Pik-2, and RRS1/RPS4 pairs against bacterial and fungal pathogens. The paired *NLR* genes are in close proximity, in a head-to-head orientation, and they share promoter sequences [132,133,134]. Such a head-to-head orientation has been described for the *Prv* and the *Fom-1* genes of *Cucumis melo* conferring resistance to the papaya ring spot virus and to some races of the *Fusarium oxysporum* fungus [97]. Whether this pair of TNLs encoding genes and other resistance genes to viruses fully fits the paired integrated decoy model will need further investigations.

Another way for two NLRs to function in a common resistance pathway is the sensor/helper mode where the sensor detects the Avr and transduces the signal to the helper NLR [131,132,135]. The helpers, which belong to the RNL class of NLRs (Figure 1B), act downstream of the sensor with no physical link detected so far. Moreover, unlike paired NLRs, sensor and helper coding genes are not located at the same locus. In most angiosperms, these helper RNLs are encoded by two gene families [136], *Activated disease resistance 1* (*ADR1*) and *N requirement gene 1* (*NRG1*). In the plant family Solanaceae, helper NLRs are of the NRC (NLR required for cell death) clade, such as NRC2, which acts downstream of the Rx NLR [137]. 

**Figure 4:**
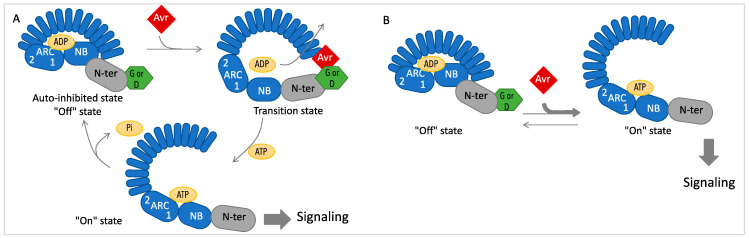
(**A**). In the classical switch model, intramolecular interactions of NB-LRR domains maintain the protein in an “off” state in the absence of the elicitor (Avr). In the presence of Avr, the exchange of ADP for ATP allows the NB-ARC domain to change conformation and protein to reach an “on” state inducing downstream signaling. A rapid reset of intramolecular interactions is needed to allow repeated rounds of recognition or limited signaling. In the *N*-encoded NB-LRR, the LRR domain is involved in the specific recognition of the Avr and the N-terminal TIR domain interacts with the NRIP1 cofactor. (**B**). An alternate model suggests an equilibrium between the “off” and “on” states and that Avr binding to the “on” state shifts the equilibrium toward the signaling competent form [138].

Whether working as singletons or in pairs, R proteins need to be tightly repressed in the absence of effectors due to their death-inducing function. One of the numerous regulation points is at the protein level [138,139]. This includes intermolecular interactions in paired NLRs, where the sensor interacts with the executor to repress it. In the singleton NLRs (Figure 4), intramolecular interactions between the domains ensure an auto-inhibited conformation known as the “Off” state where the nucleotide-binding pocket of the NB-ARC domain is closed and bound to ADP. Upon pathogen recognition, an “On” active state is favored with an opening of the binding pocket and ADP exchange for ATP. This molecular switch model has been confirmed by structure–function studies for several R proteins including antiviral examples [140,141,142]. This limits the modifications that can be made in the R proteins to broaden their pathogen recognition range. An example is the single D to V substitution of the MHD motif of the ARC domain that usually results in an autoactivated protein due to its preference for ATP binding [143,144].

## 4. Important Advances in the Comprehension of HR

### 4.1. The Continuum of Resistance: Uncoupling Cell Death and Resistance

**Figure 5:**
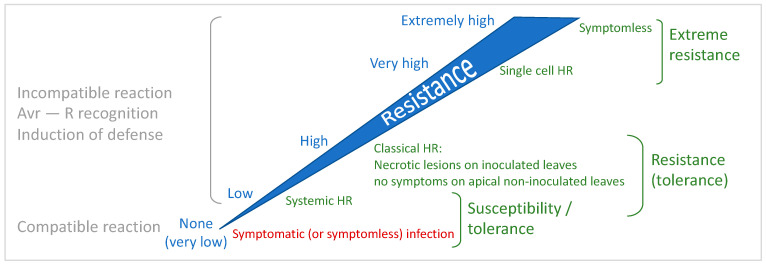
Outcomes of plant–virus interactions can be considered a continuum of host responses from extremely efficient defense resulting in virus restriction without cell death to no apparent defense, through cell death and virus spread limited to single or few cells and trailing cell death resulting in systemic necrosis with partial or no virus restriction [33]. Total or single cell restriction of the virus is viewed as extreme resistance as the virus is not easily detected and no macroscopic symptoms are observed. Plants presenting a classical HR (or weak systemic HR) can be considered resistant or tolerant according to the level of virus restriction and symptoms. Plants developing systemic HR are often viewed as susceptible even if they partially limit the virus titer due to the dramatic phenotype they present. Asymptomatic plants developing a compatible reaction meet the criteria of tolerance and susceptibility. The systemic spread of the virus in a plant does not mean that there is no resistance at all, as mutations in the salicylic pathway or in a viral effector can increase viral titer [162]. The left side of the scheme presents the outcome of the gene-for-gene relation as originally conceived by Flor [47].

Due to an increasing number of papers relating that resistance and PCD, the two components of HR, can be physiologically, genetically, and temporally uncoupled, Künstler and colleagues proposed to consider HR as a combination of these two components with varying contributions [33]. These relative contributions change according to the level of R and Avr product expression, the presence of a dysfunctional PCD regulator, or according to the timing and speed and therefore effectiveness of the resistance responses. This leads to an array of outcomes ranging from a (macroscopically) symptomless extreme resistance to an inefficient HR resulting in systemic HR (SHR or HLR for hypersensitive-like response) or to complete susceptibility (Figure 5). As a consequence, the appearance of necrosis on inoculated or non-inoculated apical leaves (phenotype) should be regarded carefully; if a link with HR (resistance mechanism) can be established at the physiological, molecular, or biochemical level, it should be called HR or SHR/HRL, whereas in the absence of knowledge or any HR marker, the term “necrosis” or “systemic necrosis” should apply (Figure 5). Necrosis with no link to resistance has been described for some CMV isolates in many Arabidopsis ecotypes where it is attributable to protein 1a [145]. On the contrary, if markers of the typical HR are observed, then the designation HLR is much more appropriate [105,114,146].

The decrease in resistance efficiency was estimated under HR-inducing and SHR-inducing conditions compared to a compatible reaction in the RCY1-CMV pathosystem, and SHR was suggested to represent a resistance mechanism at the plant population level rather than at the individual level [147,148]. This view of a continuum of resistance has been followed or confirmed in several virus–plant interactions [146,149,150,151,152,153,154] and has allowed the study of ER, HR and SHR as belonging to a common process with common genes and pathways [66,155,156].

If the transition from incompatible to compatible reactions is viewed as a continuum, it raises the question of the continuity of necrotic to non-necrotic symptoms. This question remains largely unanswered, although some observations could suggest such a continuity as only small changes in a protein can dramatically change the phenotype of infected plants from systemic mosaic to necrosis, or from systemic vein clearing to no symptoms. Thus, a single amino acid substitution of the CMV 1a protein was responsible for changing necrotic symptoms to systemic mosaic and vice versa on several *Nicotiana* species [157]. Similarly, a single amino acid substitution was sufficient to change a symptomatic to an asymptomatic GFLV [158]. A link has also been established between six cysteine rich carlavirus VSRs, and either necrosis or leaf malformations in *N. occidentalis* [159]. In addition, mutants with a single amino acid addition in a comovirus helicase, capable of inducing HR-type PCD upon transient expression in *N. benthamiana*, were shown to either compromise or accelerate cell death [160]. Recently, a single amino acid substitution in the MP of tomato mosaic virus was associated with Tm-2^2^ resistance breaking and systemic necrosis [161]. Whether these different outcomes induced by variations in the viral pathogenicity determinant really reflect a continuity in the symptoms needs to be further investigated. 

### 4.2. Resistosomes, NLR Networks, and Convergence

**Figure 6:**
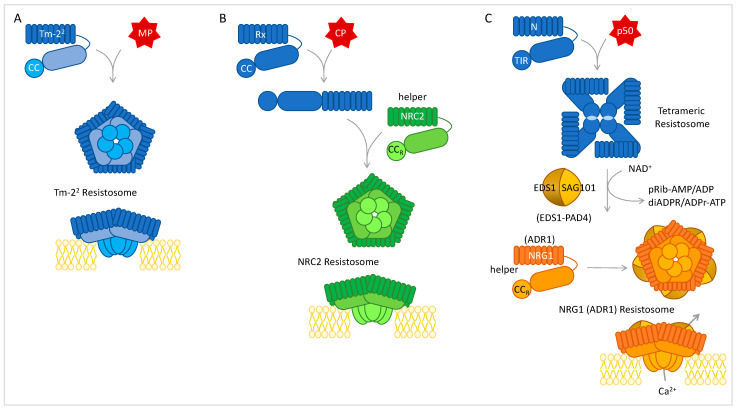
Oligomerization and localization at the plasma membrane are required for different NLR mediated cell death and defense reactions. (**A**). The tomato Tm-2^2^ CC-NLR has been shown to oligomerize and localize at the plasma membrane upon tobamovirus MP recognition. The CC domain induces cell death when tethered at the membrane [172]. (**B**). When activated by the PVX-CP, the Rx CC-NLR triggers oligomerization of NRC2 at the membrane without forming a stable complex with it. A channel activity has not, however, been demonstrated [137]. (**C**). TIR-NLRs such as N assemble four mers to form a holoenzyme with NAD hydrolase activity. The signaling nucleotide-derived small molecules induce an EDS1-SAG101 (alternatively an EDS1-PAD4 for other TNLs) dependent hetero-oligomeric resistosome of NRG1 (alternatively ADR1) at the plasma membrane and display a Ca^2+^-permeable channel activity leading to hypersensitive cell death [170,171].

PRRs in PTI and NLRs in ETI, although differing in their structure and cellular localization, share common downstream responses such as cell wall fortification, ion fluxes, ROS and nitric oxide (NO) burst, phytohormone synthesis, lipid peroxidation, and transcriptional reprograming [151,163,164]. However, they diverge in the timing and intensity of the resulting defense (Figure 2), and most importantly, only ETI leads to PCD during HR (or HLR) [26]. The recent discovery of plant resistosomes has greatly accelerated our comprehension of NLR-induced reactions and PCD. In 2019, Wang and collaborators showed that the activated, ATP-bound form of the CC-NLR ZAR1 sensing a *Xanthomonas campestris* Avr adopted a pentameric wheel-like structure with the five very N-terminal α-helices protruding and forming a funnel-shaped structure required for plasma membrane (PM) association and PCD induction [165]. Due to its function in resistance, this ATP-induced oligomer was called a resistosome to make a parallel with animal apoptosomes and inflammasomes. This resistosome was shown to exhibit a cation-permeable channel activity [166]. In the following year, two TIR-NLRs conferring resistance to an oomycete [167] and a bacterium [168] were shown to assemble into tetrameric ATP-bound resistosomes upon cognate Avr recognition, and form two active nicotinamide adenine dinucleoside (NAD) hydrolase sites (each made of a dimer of TIR domains) involved in HR establishment. Although a tetrameric structure was not demonstrated for the tobacco N protein, its oligomerization in the presence of its Avr was demonstrated [169]. Helper NLRs of the ADR1 and NRG1 families also form resistosomes at the PM and display Ca^2+^-permeable channel activity [170,171] (Figure 6). The Tm-2^2^ tomato protein conferring resistance to tobamoviruses was shown to self-associate at the PM after recognition of the viral MP, where it induces cell death [172] (Figure 6). The Rx CC-NLR, on the other hand, does not oligomerize but was shown to induce the oligomerization of its helper NRC2 which accumulates at the membrane [137] (Figure 6). Altogether, a convergent working model emerges where a homo or hetero-oligomer forms at the PM and induces a Ca^2+^ influx causing cell death [173]. 

Although R-Avr recognition is specific (often race specific), plant defenses are known to be non-specific and provide a broad-spectrum resistance. This can be partly due to the complex networks sensors can establish with helper NLRs; some hNLRs are redundant and required for several sensors, as exemplified by NRC2, NRC3, and NRC4, which redundantly contribute to the immunity mediated by several sensors including Rx and Sw5 [56] or by NRG1 and ADR1, which are required for all known TNL signaling [174,175,176]. These complex networks are thought to increase evolvability while maintaining defense robustness. They also avoid the need for specific pathways against each pathogen [176].

### 4.3. Subcellular Localization

The localization or relocation of sensor or helper NLRs forming resistosomes to the PM is a crucial requirement for PCD. However, like for other pathosystems, a nuclear localization of proteins conferring resistance to viruses is essential for effective host defense and/or PCD [177]. EDS1 (Enhanced disease susceptibility 1) in complex with SAG101 (Senescence-associated gene 101) plays a key role in the TNL induction of resistance, not only in the cytoplasm to form helper resistosomes, but also in the nucleus for transcriptional reprogramming and resistance [178,179]. After tobamovirus p50 and NRIP1 recognition, the N receptor associates, possibly as a dimer, with the transcription factor (TF) SPL6 (Squamosa Promoter Binding Protein Like 6) in the nucleus [180]. This TF is required for restricting the virus. N-mediated resistance also depends on NPR1 (non-expressor of pathogenesis-related genes 1), a receptor of salicylic acid (SA) which enters the nucleus and controls the majority of SA-dependent genes by interacting with TGA TFs [181,182]. 

Moreover, CNLs shuttle between the cytoplasm and the nucleus to ensure complete immunity. Thus, a proper localization of Rx in the nucleus is necessary for efficient restriction of PVX and translational arrest [183], a common host antiviral response [184]. This partitioning is ensured by the chaperone SGT1 (Suppressor of the G2 allele of skp1) [185] and the cytoplasmic RanGap2 (RanGTPase-activating protein 2) cofactor, which retains a pool of Rx [186]. In the nucleus, Rx binds DNA in its active state, with a preference for transcription start bubbles. When complexed to GLK1, a Golden 2-like TF, it drives its sequence specificity [187]. Recently a bromodomain-containing protein (DBCP) and an RNA-binding protein (GRP7) were implicated in the nuclear regulation of the Rx accumulation level and Rx-mediated defenses [154,188]. 

The nucleocytoplasmic distribution of Sw5-b was recently associated with different roles in the defense against TSWV; the cytoplasmic Sw5-b was responsible for cell death to inhibit the virus replication, whilst the nuclear Sw5-b was involved in a weak inhibition of replication but a strong hindrance of both cell-to-cell and long-distance movements [189]. How the nuclear Sw5-b induces this layer of defense remains unknown. Similarly, the Sw5-a protein, conferring resistance to a begomovirus, has a dual nucleus and PM distribution [190]. Even though cell death predominantly results from cytoplasmic events, it seems to be under the control of nuclear actors such as the WRKY1 TF, which were shown to regulate HR cell death induced by two geminiviruses belonging to different genera [116,191]. Moreover, the RepA nuclear localization is required for the cell-death induction [116]. 

Other subcellular localizations might be important for sensing the virus and inducing resistance, as exemplified by the Rsc4-3 CNL, which locates in the cell wall and needs palmitoylation to induce HR upon recognition of the Avr CI directly in the apoplast [70].

### 4.4. HR Is Controlled by UPS and Autophagy

In recent years, progress has been made in understanding HR regulation by ubiquitin (Ub) and the 26S ubiquitin-proteasome system (UPS). In 2019, a formal link was established between the N-terminal Ub fused ribosomal protein Ub extension protein 1 (UEP1) and cell death and resistance. A reduced expression of UEP1 led to increased ROS and PR protein levels, cell death, and resistance to TMV and CMV [192]. UPS was also demonstrated to regulate the turn-over of positive (NPR1) and negative (NPR3/4) regulators of SA-dependent genes [182,193]. UPS is also involved in the degradation of stress proteins including NLRs, EDS1, and WRKY TFs in SA-induced NPR1 condensates (SINCs), promoting cell survival during HR [194]. Finally, deubiquitinases have been shown to play a critical role in limiting HR and cell death [195,196].

Autophagy also limits HR-related PCD through SA and ROS modulation [197], as demonstrated in TMV-infected tomatoes [198]. Conversely, ROS favor autophagy through disrupting the interaction between ATG3 and a cytosolic GAPDH (GAPC) during *N*-mediated resistance to TMV [199]. Recently, autophagy was also found to be involved in SA-dependent lignin deposition and cell-wall reinforcement during bacterium-induced HR [200]. Many viral proteins interact with autophagy [201]; some interact with GAPCs, resulting either in an increase or decrease in autophagy [202,203,204]. Others intervene at later stages such as vacuole acidification [205]. Although this generally amounts to combating a defense mechanism by inducing the degradation of antiviral factors (such as proteins of the RNAi or autophagy pathways), it is not always quite clear whether these interactions benefit the plant or the virus [206]. More studies will be required to grasp these complex interplays.

Vacuolar cysteine proteases known as vacuolar processing enzymes (VPEs), although presenting low sequence similarity, are related to caspases [207]. They were shown almost twenty years ago to play a critical role in N-mediated PCD through the induction of vacuole collapse [208]. How VPEs are connected to NLRs or autophagy-related regulation of PCD needs to be investigated. For example, a link (direct or indirect) between VPEs and resistosomes could be explored. This should be undertaken in the context of a viral induction of HR, as the role of VPEs in PCD is suggested to be pathosystem dependent [207].

## 5. Conclusions

A lot has been learnt in the recent years about HR and how specific interactions can lead to broad resistance through networks. However, the different pathosystems also revealed specific requirements, such as those of Sw5-b, which does not rely on EDS1 nor NPR1 [181]. Although proteins interacting with NLRs have been identified [131], their role in the different mechanisms and their regulation needs to be further understood and more antiviral NRLs need to be cloned and studied to elaborate a clearer picture. Of course, the identification of antiviral RLPs and their cognate PAMPs would be of great interest to the community.

The recent advances in the knowledge of the NLRs have opened the way to their engineering in view to broadening or changing their specificity of recognition. Thus, engineering of the ID of an ID-NLR sensor working in a pair with an executer has proved effective at recognizing a new effector. This was achieved by a few substitutions in the ID or by replacing it with a nanobody coding sequence [209,210]. An autoactivated mutant of Pvr9 was also exploited to detect a viral protease by fusing its coding sequence to the cleavage site of the potyvirus NIa and a tag. The tag inhibited the NLR auto-activity that was recovered in the presence of the viral protease [211]. More recently, a single substitution was introduced in the helper NRC2 to evade its binding to a virulence factor blocking its oligomerization into a resistosome. This engineered NRC2 had a restored activity [212]. These few examples illustrate how our knowledge can be exploited to help the plants in their arms race against pathogens.

## Figures and Tables

**Table 1:** Cloned genes conferring resistance to plant viruses.

Year	ER/HR	Plant of Resistance Origine	Resistance Gene(s)/Locus	Type of NLR	Virus (*Genus*; *Family*)	Viral Determinant	References	Cloning Strategy	Identification of Cognate Avr	Confirmation of R Function
1995	HR	Tobacco *Nicotiana glutinosa*	*N*	TIR-NBS-LRR	Tobacco mosaic virus = TMV (*Tobamovirus*; *Virgaviridae*)	130K Replicase (p50 helicase domain)	[50] [78]	Transposon tagging	Chimeric viruses	Transgenic expression
1999	ER (SHR in transgenic *N. benthamiana*)	Wild potato *Solanum andigena*	*Rx*-*1* (Chr XII)	CC-NBS-LRR	Potato virus X = PVX (*Potexvirus*; *Alfaflexiviridae*)	CP	[79] [80]	Map-based cloning	Chimeric viruses (TMV vector expressing PVX CP)	Transgenic expression
2000	ER (HR under weak promoter in tobacco)	Potato *Solanum acuale*	*Rx-2* (Chr V)	CC-NBS-LRR	PVX (*Potexvirus*; *Alfaflexiviridae*	CP	[71] [81]	Agrobacterium-mediated expression of a library of *Rx*-homologues	Point mutations in CP gene	Transgenic expression
2000	HR	Thale cress *Arabidopsis thaliana*	*HRT*	CC-NBS-LRR	Turnip crinkle virus = TCV (*Betacarmovirus*; *Tombusviridae*	CP	[82]	Map-based cloning	Transgenic expression	Transgenic expression
2001	ER	Tomato *Solanum peruvianum*	*Sw5-b*	CC-NBS-LRR	Tomato spotted wilt virus = TSWV and other tospoviruses (*Orthotospovirus*; *Tospoviridae*)	Nsm (MP)	[83] [84]	Map-based cloning Bac screening	Chimeric viruses (AlMV vector with MP of TSWV)	Transgenic expression
2002	HR	Thale cress *Arabidopsis thaliana*	*RCY1* same locus as *HRT*	CC-NBS-LRR	Cucumber mosaic virus = CMV (*Cucumovirus*; *Bromoviridae*)	CP	[85] [86]	Map-based cloning	Chimeric viruses	Transgenic expression
2002	ER to PVA & PVV? Systemic necrosis to PVY	Potato *Solanum tuberosum*	*Y-1* (Chr XI)	TIR-NBS-LRR	Potato virus Y = PVY, potato virus A = PVA, potato virus V = PVV (*Potyvirus*; *Potyviridae*)	?	[87]	Homology cloning	-	Transgenic expression
2003	ER	Tomato *Solanum peruvianum*	*Tm-2* (alleles *Tm-2* & *Tm-2^2^*)	CC-NBS-LRR	Tomato mosaic virus = ToMV & TMV (*Tobamovirus*; *Virgaviridae*)	30 K MP (C-term)	[67] [88]	Transposon tagging Homology cloning (*Tm-2^2^*)	Deletion of C-terminus of MP in virus Transgenic expression	Transgenic expression
2006	SHR in transgenic *N. benthamiana* (ER in *P. vulgaris*?)	Common bean *Phaseolus vulgaris*	*PvCMR1* (*RT4-4*)	TIR-NBS-LRR	CMV (*Cucumovirus*; *Bromoviridae*)	2a	[89]	Homology cloning	Transient expression (agrobacterium)	Transgenic expression
2007	HR	Common bean *Phaseolus vuulgaris*	*PvVTT1*	TIR-NBS-LRR	Bean dwarf mosaic virus = BDMV (*Begomovirus*; *Geminiviridae*)	BV1 (NSP)	[90] [91]	cDNA substraction & cloning	Chimeric viruses	Transgenic expression
2010	HR	Chinese cabbage *Brassica campestric*	*BcTuR3* (cloned but not confirmed)	TIR-NBS-LRR	Turnip mosaic virus = TuMV (*Potyvirus*; *Potyviridae*)	?	[92]	Homology cloning	-	Not confirmed
2011	HR	Pepper *Capsicum* spp.	*L* (alleles *L^2^*–*L^4^*)	CC-NBS-LRR	TMV, ToMV, paprika mild mottle virus = PaMMV, peper milds mottle virus = PMMV (*Tobamovirus*; *Virgaviridae*)	CP	[74] [93]	Map-based (*L^3^*) andhomology-based cloning (*L^1^*, *L^2^*, *L^4^*)	Chimeric viruses & mutants	Transient expression (agrobacterium)
2012	HR	White tobacco *Nicotiana sylvestris*	*N*′	CC-NBS-LRR	TMV & other tobamoviruses (*Tobamovirus*; *Virgaviridae*)	CP	[75] [94]	Homology cloning (L)	Point mutations in CP gene	Transient expression (agrobacterium)
2012	HR	Black gram *Vigna mungo*	*CYR1*	CC-NBS-LRR	Mungbean yellow mosaic India virus = MYMIV (*Begomovirus*; *Geminiviridae*)	AV1 (CP)?	[95]	Map-based cloning	*In silico* model	Not confirmed
2013	ER	Melon *Cucumis melo*	*Prv*	TIR-NBS-LRR	Papaya ringspot virus = PRSV (*Potyvirus*; *Potyviridae*)	?	[96] [97]	Map-based cloning BAC screening	-	Genome editing
2014	HR	Pepper	*Pvr9* (Chr VI)	CC-NBS-LRR	Pepper mottle virus = PepMoV (*Potyvirus*; *Potyviridae*)	NIb (Pol)	[72] [73]	Agrobacterium-mediated expression of a library of *R* candidates	Transient expression (agrobacterium)	Transient expression (agrobacterium)
2014	ER	Turnip *Brassica rapa*	*TuRBO7* (1 candidate gene, not identified, not cloned)	CC-NBS-LRR	TuMV (*Potyvirus*; *Potyviridae*)	?	[98]	Map-based cloning	-	Not confirmed
2016	ER (SHR with strain SMV-G7)	Soybean *Glycine max*	*Rsv1-h* (2 candidate genes identified, not cloned)	CC-NBS-LRR	Soybean mosaic virus = SMV (*Potyvirus*; *Potyviridae*)	P3 & HC-Pro	[99] [100]	Map-based cloning	Chimeric viruses & mutants	Not confirmed
2017	ER / HR	Pepper *Capsicum annuum*	*Pvr4* / *Pvr7* (ChrX)	CC-NBS-LRR	PepMoV, PVY (*Potyvirus*; *Potyviridae*)	NIb (Pol)	[76] [101] [102]	Map-based cloning BAC screening	Transient expression (agrobacterium)	Transient expression (agrobacterium)
2017	HR	Pepper *Capsicum annuum*	*Tsw* (Chr X) same locus as *Pvr4*	CC-NBS-LRR	TSWV (*Orthotospovirus*; *Tospoviridae*)	NSs (VSR)	[76] [103]	Map-based cloning BAC screening	Transient expression (agrobacterium)	Transient expression (agrobacterium)
2017	HR	Sugar beet *Beta vulgaris*	*Rz2*	CC-NBS-LRR	Beet necrotic yellow vein virus = BNYVV (*Benyvirus*; *Benyviridae*)	TGB1 (MP)	[104] [105]	Mapping-by-sequuencing	Transient expression (agrobacterium)	Silencing by hairpin in transgenic sugarbeet
2018	ER / HR	Wild tomato *Solanum habrochaites*	*Ty-2* = *TYNBS1*	CC-NBS-LRR	Tomato yellow leaf curl virus = TYLCV (*Begomovirus*; *Geminiviridae*)	Rep / C1 (replication-associated protein)	[77] [106]	Map-based cloning and transgenic expression	Transient expression (agrobacterium)	Transgenic expression
2020	ER	Wild potato *Solanum stoloniferum*	*Ry_sto_* = *Ry-f_sto_*	TIR-NBS-LRR	PVY, PVA (*Potyvirus*; *Potyviridae*)	CP / previously NIa involved	[66] [107]	Enrichment sequencing & Pac Bio single-molecule real-time sequencing (SMRT RenSeq)	Transient expression (agrobacterium)	Transient expression (agrobacterium)
2021	HR	Tomato *Solanum lycopersicum*	*Sw5-a*	CC-NBS-LRR	Tomato leaf curl New Dehli virus = ToLCNDV (*Begomovirus*; *Geminiviridae*)	AC4 (VSR, suppressor of cell death)	[108]	miRNAomics identified a regulator of *Sw5-a*	Identification of Sw5-a interactant Transient expression (agrobacterium)	Virus induced gene silencing
2021	ER	Soybean *Glycine max*	*Rsc 4-3* = *Rsv 3* / *NBS_C*	CC-NBS-LRR	SMV (*Potyvirus*; *Potyviridae*)	CI (cylindrical inclusion protein, replication & movement)	[70]	Fine mapping Transient expression (agrobacterium) Cas9-assisted mutation of candidate genes	Chimeric viruses & transient expression (agrobacterium)	Transient expression (agrobacterium) & genome editing
2022	ER	Wild potato *Solanum chacosense*	*Ry_chc_* (ChriX)	TIR-NBS-LRR	PVY (*Potyvirus*; *Potyviridae*)	?	[109]	Map-based cloning	-	Transgenic expression

?: not identified or not confirmed, CP = coat protein, MP = movement protein, NSP = nuclear shuttle protein, NIb = nuclear inclusion b, Pol = polymerase, HC-Pro = helper component-protease, VSR = viral suppressor of RNA silencing.
